# Sequencing *Mycobacteria* and Algorithm-determined Resistant Tuberculosis Treatment (SMARTT): a study protocol for a phase IV pragmatic randomized controlled patient management strategy trial

**DOI:** 10.1186/s13063-022-06793-w

**Published:** 2022-10-08

**Authors:** Annelies Van Rie, Elise De Vos, Emilyn Costa, Lennert Verboven, Felex Ndebele, Tim H. Heupink, Steven Abrams, SMARTT team, Noriah Maraba, Heeran Makkan, Trevor Beattie, Zandile Rachel Sibeko, S’thabiso Bohlela, Pulane Segwaba, Emmanuel Ayodeji Ogunbayo, Nomadlozi Mhlambi, Felicia Wells, Leen Rigouts, Gary Maartens, Francesca Conradie, John Black, Sam Potgieter, Boitumelo Fanampe, Anneke Van der Spoel Van Dyk, Salome Charalambous, Gavin Churchyard, Rob Warren

**Affiliations:** 1 Family Medicine and Population Health, Faculty of Medicine and Health Sciences, University of Antwerp, Antwerp, Belgium; 2 South African Medical Research Council Centre for Tuberculosis Research, DST NRF Centre of Excellence for Biomedical Tuberculosis Research, Division of Molecular Biology and Human Genetics, Faculty of Medicine and Health Sciences, Stellenbosch University, Cape Town, South Africa; 3 Aurum Institute, Johannesburg, South Africa; 4 Free State Department of Health, Bloemfontein, South Africa; 5 Universitas Academic Laboratory, National Health Laboratory Service and Department of Medical Microbiology, Faculty of Health Sciences, University of the Free State, Bloemfontein, South Africa

**Keywords:** Drug resistance, Tuberculosis, Whole-genome sequencing, Clinical trial, Pragmatic, Strategy trial, Treatment recommender

## Abstract

**Background:**

Rifampicin-resistant tuberculosis (RR-TB) remains an important global health problem. Ideally, the complete drug-resistance profile guides individualized treatment for all RR-TB patients, but this is only practised in high-income countries. Implementation of whole genome sequencing (WGS) technologies into routine care in low and middle-income countries has not become a reality due to the expected implementation challenges, including translating WGS results into individualized treatment regimen composition.

**Methods:**

This trial is a pragmatic, single-blinded, randomized controlled medical device trial of a WGS-guided automated treatment recommendation strategy for individualized treatment of RR-TB. Subjects are 18 years or older and diagnosed with pulmonary RR-TB in four of the five health districts of the Free State province in South Africa. Participants are randomized in a 1:1 ratio to either the intervention (a WGS-guided automated treatment recommendation strategy for individualized treatment of RR-TB) or control (RR-TB treatment according to the national South African guidelines). The primary effectiveness outcome is the bacteriological response to treatment measured as the rate of change in time to liquid culture positivity during the first 6 months of treatment. Secondary effectiveness outcomes include cure rate, relapse rate (recurrence of RR-TB disease) and TB free survival rate in the first 12 months following RR-TB treatment completion. Additional secondary outcomes of interest include safety, the feasibility of province-wide implementation of the strategy into routine care, and health economic assessment from a patient and health systems perspective.

**Discussion:**

This trial will provide important real-life evidence regarding the feasibility, safety, cost, and effectiveness of a WGS-guided automated treatment recommendation strategy for individualized treatment of RR-TB. Given the pragmatic nature, the trial will assist policymakers in the decision-making regarding the integration of next-generation sequencing technologies into routine RR-TB care in high TB burden settings.

**Trial registration:**

ClinicalTrials.gov NCT05017324. Registered on August 23, 2021.

## Administrative information

Note: the numbers in curly brackets in this protocol refer to the SPIRIT checklist item numbers. The order of the items has been modified to group similar items (see http://www.equator-network.org/reporting-guidelines/spirit-2013-statement-defining-standard-protocol-items-for-clinical-trials/).

**Table:** 

Title {1}	Sequencing Mycobacteria and Algorithm-determined Resistant Tuberculosis Treatment (SMARTT): a study protocol for a phase IV pragmatic randomized controlled patient management strategy trial.
Trial registration {2a and 2b}.	Registered on clinical trials.gov register number NCT05017324 on August 23, 2021, https://clinicaltrials.gov/ct2/show/NCT05017324
Protocol version {3}	Protocol version 6; June 9, 2021
Funding {4}	FWO TBM (Applied Biomedical Research with a Primary Social finality) grant number T001018N and FWO Odysseus grant number G0F8316N.
Author details {5a}	Annelies Van Rie^1*^, Elise De Vos^1^, Emilyn Costa^2^, Lennert Verboven^1^, Felex Ndebele^3^, Tim H Heupink^1^, Steven Abrams^1^, SMARTT team, Boitumelo Fanampe^4^, Anneke Van der Spoel Van Dyk^5^, Salome Charalambous^3^, Gavin Churchyard^3^, Rob Warren^2^. ^1^Family Medicine and Population Health, Faculty of Medicine and Health Sciences, University of Antwerp, Antwerp, Belgium; ^2^South African Medical Research Council Centre for Tuberculosis Research, DST NRF Centre of Excellence for Biomedical Tuberculosis Research, Division of Molecular Biology and Human Genetics, Faculty of Medicine and Health Sciences, Stellenbosch University, Cape Town, South Africa; ^3^Aurum Institute, Johannesburg, South Africa; ^4^Free State Department of Health, Bloemfontein, South Africa; ^5^Universitas Academic Laboratory, National Health Laboratory Service and Department of Medical Microbiology, Faculty of Health Sciences, University of the Free State, Bloemfontein, South Africa. *Corresponding author: Annelies Van Rie (annelies.vanrie@uantwerpen.be)
Name and contact information for the trial sponsor {5b}	University of Antwerp Contact Annelies.vanrie@uantwerpen.be
Role of sponsor {5c}	SMARTT is an investigator-initiated research trial with the University of Antwerp acting as the study sponsor. The Aurum Institute acts as the Clinical Research Organization responsible for the coordination of the field work in South Africa. The University of Stellenbosch houses the research laboratory for the trial. The principal and associate investigators are solely responsible for the conception, execution, analysis, and dissemination of the research work.

## Introduction

### Background and rationale {6a}

#### Background

The occurrence of 500,000 cases of rifampicin-resistant tuberculosis (RR-TB) annually threatens TB control and global efforts to reach the targets of the World Health Organization (WHO) End TB Strategy [1]. RR-TB treatment is complex, lengthy, often associated with side effects, and costly from the patient, health care provider, and health care system perspective. This results in poor treatment outcomes, high death rates, and continued transmission of drug-resistant TB in the community and health care settings [2].

Great progress in RR-TB management has been made in the past decade. The WHO endorsement of the first-line molecular line probe assays (LPA) in 2008 resulted in rapid diagnosis of resistance to rifampicin (RIF) and isoniazid (INH) in people at high risk of multidrug-resistant TB (MDR-TB) [3]. Endorsement of the Xpert MTB/RIF [4] assay in 2011 and Xpert Ultra assay in 2017 [5] as the initial test for patients presenting with symptoms of TB has revolutionized RR-TB diagnosis and reduced the diagnostic RR-TB gap, from 7% of patients with bacteriologically confirmed TB being tested for RIF resistance in 2012 to 71% in 2020 [1, 6].

The approval of bedaquiline (BDQ) for treatment of RR-TB enabled the replacement of the lengthy (15 to 18 months) treatment with a shorter (9–12 month) all-oral treatment regimen. Since 2019, a 7-drug regimen including BDQ, a fluoroquinolone (FQ), ethionamide (ETO), ethambutol (EMB), high dose INH, pyrazinamide (PZA) and clofazimine (CFZ) is recommended as the regimen of choice for most patients diagnosed with RR-TB [7]. For patients who previously received treatment for RR-TB or in whom FQ resistance is diagnosed, a longer all-oral regimen [BDQ, linezolid (LZD), a FQ, CFZ and/or cycloserine or terizidone (TZD), with or without additional drugs] can be used. In August 2019, pretomanid (Pa) was approved by the United States Food and Drug Administration in combination with BDQ and LZD for patients with FQ resistance who have not been exposed to these drugs [8].

Although these regimens greatly improve treatment outcomes of patients suffering from RR-TB [9–11], this one-size-fits-all approach could contribute to the amplification of resistance, followed by transmission of drug-resistant strains [12]. As Acquah and Furin wrote “If we are serious about ending TB, we need to do things differently. And this means simplicity cannot be the goal” [13]. To achieve maximal patient benefit and to protect the long-term effectiveness of the novel RR-TB treatment regimens, detailed drug susceptibility testing (DST) at the individual level is needed. The WHO recommends that RR-TB patients should receive at least 4 effective drugs as this has been associated with improved treatment outcomes [7]. This could then in turn reduce the risk of drug resistance amplification, limit the development of very hard-to-treat TB, and lessen the risk of transmission of highly drug-resistant TB. Unfortunately, DST is currently a complex, lengthy, multi-step process that requires specialized skills and laboratory infrastructure to perform PCR-based tests and *Mycobacterium tuberculosis* (*Mtb*) culture. Due to these hurdles, treatment for RR-TB continues to focus on empiric regimens, where treatment is prescribed in the absence of knowledge of the drug resistance profile of the *Mtb* strain.

Whole genome sequencing (WGS) can identify the presence of variants in all known candidate resistance genes in a single procedure. In 2018, the WHO endorsed next-generation sequencing (NGS), which includes both whole genome and targeted sequencing, given its potential for rapid diagnosis of drug-resistant TB in diverse clinical laboratory settings worldwide. NGS overcomes many challenges associated with conventional phenotypic testing as well as the limitations of less comprehensive molecular tests [14]. The uptake of NGS for DST has been hindered by concerns regarding the sensitivity and specificity of NGS/WGS for second-line drugs, the challenges in translating WGS results into individualized treatment regimen composition, the cost and human resource requirements, and the expected implementation challenges in high TB burden settings [12, 14].

In this paper, we outline the background and rationale for the Sequencing Mycobacteria and Algorithm-determined Resistant Tuberculosis Treatment (SMARTT) trial and describe the design of the pragmatic single-blinded randomized controlled medical device trial in accordance with the *Standard Protocol Items: Recommendations for Interventional Trials* (SPIRIT) [15].

#### Rationale

##### Drug-susceptibility testing of *Mycobacterium tuberculosis*: current practice

Even though the WHO emphasizes the importance of DST in guiding RR-TB treatment decisions [7], most RR-TB patients receive an empiric treatment regimen solely based on the presence of resistance to RIF and drug exposure history. The tools to perform DST upon RR-TB diagnosis currently used in routine care include first-line LPA to detect resistance to RIF and INH by evaluating the presence of a number of mutations in *rpoB* and *katG* genes, and *inhA* promoter region [3], a second-line LPA to detect resistance to aminoglycosides (*eis* and *rrs* genes), and FQ (*gyrA* and *gyrB* genes) [16], and culture-based phenotypic testing for other drugs.

The WHO recommends DST for INH in all RR-TB patients and assessment of FQ resistance for all MDR-TB patients, this is rarely implemented in high-burden countries [1]. In 2016, only 36% of patients with RR-TB globally received DST for second-line drugs. In a few settings, such as South Africa, the national guidelines recommend that all people presenting with symptoms or signs of TB are assessed by the Xpert Ultra assay, that first-line LPA is performed in all people diagnosed with RR-TB, FQ resistance is evaluated in patients with confirmed MDR-TB, and phenotypic testing of BDQ and LZD is performed in all patients with FQ resistance [17]. Nevertheless, a study in three provinces in South Africa showed that under routine conditions, only 63% of RR-TB cases were assessed for INH resistance, and only 65% of MDR-TB cases were evaluated for the presence of FQ resistance [18].

DST for ETO, EMB, PZA, CFZ, LZD, TZD, delamanid (DLM), and Pa is rarely determined in any high RR-TB burden setting. Consequently, the presence of four effective drugs in the current standard all-oral 7-drug empiric treatment regimen is rarely confirmed for any RR-TB patient residing in high MDR-TB burden settings.

To improve rapid molecular DST, the WHO recently endorsed the Xpert MTB/XDR assay which can detect resistance to INH, ETO, FQs and second-line injectable drugs directly on a sputum specimen by investigating a number of variants in eight drug resistance-conferring genes [19]. While this expands the reach of current rapid tests, the assay still lacks the ability to detect mutations in other candidate resistance genes and cannot assess resistance to BDQ and LZD, two of the three core drugs for RR-TB treatment.

##### Whole genome sequencing for drug susceptibility testing of *Mycobacterium tuberculosis*

WGS analyses the entire *Mtb* genome, thus allowing the identification of all variants in all candidate resistance genes as well as variants outside the currently known targets. This offers several advantages, such as the ability to monitor the emergence of novel causal resistance mechanisms, the flexibility of investigating more targets without the need to revise a commercial assay, and the ability to perform accurate transmission dynamics analyses.

In 2021, the WHO endorsed a catalogue of mutations to guide the interpretation of genotypic DST results [20]. The catalogue classifies variants in 50 candidate resistance genes for 13 anti-tuberculosis drugs: the four first-line drugs (RIF, INH, EMB and PZA), the three second-line group A drugs [levofloxacin (LVX), moxifloxacin (MXF) BDQ and LZD], as well as CFZ, DLM, amikacin (AMK), streptomycin (STM), and ETO [20]. The catalogue is based on the analysis of over 38,000 *Mtb* isolates from 41 countries with paired WGS and phenotypic DST data. The performance of mutations (graded with confidence to be associated with resistance) to predict phenotypic drug susceptibility varied by drug. Specificity was very high (>90%) for all 13 drugs. Sensitivity was high (>80%) for RIF, INH, FQ, EMB, and STM, moderate (>70 %) for AMK, PZA, and ETH, but low for BDQ, DLM, CFZ, and LZD as few mutations could be confidently classified given the low prevalence to date of phenotypic resistance to these drugs in clinical isolates [20, 21].

##### WGS for routine DST and clinical care

Following the observation that WGS has high accuracy for DST of first-line drugs and reduces the need for phenotypic DST by 90% in low MDR-TB burden, WGS for genotypic DST has been successfully implemented in reference laboratories in several high-income countries [22, 23]. Reference laboratories of Public Health England and New York State were the first to implement WGS for routine *Mtb* DST [24, 25]. A shift to perform WGS for routine DST has since been implemented at several national reference laboratories, including the Netherlands [23], Belgium [26], and New South Wales in Australia [27].

Experience in using WGS to evaluate resistance to second-line drugs and to guide the individualization of RR-TB treatment is limited. A retrospective analysis of a patient with extensive drug-resistant (XDR)-TB in Australia showed that WGS provided superior data as compared to phenotypic DST in a much shorter timeframe [28]. A study of six suspected XDR-TB cases at a London teaching hospital demonstrated that WGS data can be available weeks before phenotypic DST and that WGS can contribute to clinical decision-making [29]. A study of 20 MDR-TB and five XDR-TB patients admitted to the Medical Clinic of the Research Center Borstel in Germany showed that treatment regimens were correctly designed in 93% of patients when using WGS results compared to phenotypic DST, and WGS-guided regimens did not contain any drugs to which phenotypic DST showed resistance [30]. A report on a South African MDR-TB patient highlighted the ability of WGS to design an effective individualized treatment regimen by a more complete understanding of the patient’s drug resistance pattern [31].

##### Translating WGS results into optimal individualized treatment regimens

In most high TB burden countries, medical doctors and nurses in charge of RR-TB patient care are not equipped with the knowledge required to translate *Mtb* WGS data into individualized treatment regimen [32]. Getting the right drugs to each patient poses an enormous challenge in these settings and difficulties in translating WGS data to patient management impede patient benefit from scientific advances. Data-driven approaches could facilitate the clinical use of *Mtb* WGS data. First, the use of a variant-calling tool, such as TB profiler, is important to identify the variants that confer drug resistance [33]. Next, the drug resistance profile of a strain has to be translated into an individualized regimen. Using data mining and machine learning methods, computational models can be generated to automatically, accurately and efficiently translate WGS *Mtb* data into individualized RR-TB treatment regimens [34]. These recommender models are highly flexible and can easily integrate new drugs or new knowledge on genotypic-genotypic associations as they become available. Software tools can be developed to create a user-friendly and intuitive tool for health care workers. The software can also be flexible when drug stock-outs occur, or patients develop drug toxicity.

##### The need for a clinical trial to assess the use of WGS and automated treatment recommendation for individualized treatment of RR-TB in a high RR-TB burden setting

Even though the cost of sequencing technologies has reduced substantially, the use of WGS remains limited to research settings and reference laboratories in high-income countries [35]. To inform global policy, a randomized clinical trial (RCT) to evaluate the use of WGS for individualized management of RR-TB in high-burden, resource-limited settings is needed.

Two trials of WGS for management of RR-TB are registered of which only one uses WGS to guide individualized treatment. The Individualized Drug-resistant TB Treatment Strategy trial in South Africa, which is recruiting since August 2017, randomizes patients to WGS-guided individualized treatment or standard of care [36]. The open-label MDR-TB Treatment Regimens for Ultra Short Therapy (TB-TRUST) trial in China, which is recruiting since June 2020, uses the WGS-determined PZA status to randomize participants to one of three standard regimens [37]. In addition, one trial of targeted sequencing is registered. The Test to Treat TB RCT in South Africa, which is not yet recruiting, aims to determine the impact of sputum-based targeted sequencing on guiding MDR-TB treatment [38].

### Objectives {7}

The primary objective is to evaluate the effect of WGS-guided individualized treatment recommendation on bacteriological response to treatment measured as the rate of change in time to culture positivity in liquid culture. We hypothesize that people randomized to the WGS DST strategy arm will experience faster time-to-culture conversion on RR-TB treatment compared to people randomized to the standard of care (SOC) DST strategy arm. Secondary effectiveness measures include cure rate on treatment and relapse rate (recurrence of RR-TB disease) and TB free survival rate in the first 12 months following RR-TB treatment completion. Exploratory objectives include safety and feasibility of the intervention and a health economics assessment from the health system and patient perspective.

### Trial design {8}

We designed a phase 4, single-blinded, randomized, parallel-group, pragmatic, medical device, superiority trial to evaluate a WGS-guided automated treatment recommendation strategy for individualized treatment of RR-TB. The trial is a pragmatic trial with 8 of 9 Pragmatic Explanatory Continuum Indicator Summary (PRECIS) domains scoring high (≥4) on the level of pragmatism (Table 1) [39]. Only the selection of microbiological response as the primary outcome (a surrogate marker for the patient-relevant outcomes of cure) scored <4. Another deviation from pragmatism is the reimbursement of participants for their time and effort related to attending the enrolment visit, collection of study sputum samples at weeks 2, 3, 5 and 6, and attending the treatment individualization visit.

**Table 1:** Nine PRECIS-2 dimensions of the level of pragmatism in a trial

Domain	Assessment	SMARTT	Score^**a**^
Eligibility	To what extent are the participants in the trial similar to those who would receive this intervention if it was part of usual care	All adults with RR-TB are eligible except for highly complicated cases (TBM, bone TB – as these would always be referred for expert advice). Patients with other forms of extrapulmonary TB but without pulmonary involvement, who could be treated using WGS-guided treatment recommendation under routine conditions, are excluded because of the inability to monitor their mycobacteriological response to treatment	4
Recruitment	How much extra effort is made to recruit participants over and above what would be used in the usual care setting to engage with patients?	Patients are identified through the standard laboratory RR-TB alerts and recruited at the time of RR-TB treatment initiation by a member of the district MDR-TB team	5
Setting	How different is the setting of the trial and the usual care setting?	The setting of the trial is all health care facilities of 4 of the 5 health care districts of the Free State province in South Africa where RR-TB treatment is initiated	5
Organization	How different are the resources, provider expertise and the organization of care delivery in the intervention arm of the trial and those available in usual care?	The organization of care is identical to usual care, with the routine care providers in charge of all patient management decisions and the use of drugs that are available through the DoH district pharmacies	5
Flexibility in delivery	How different is the flexibility in how the intervention is delivered and the flexibility likely in usual care?	The use of a central clinical committee to review the arguments of a care provider who disagrees with the WGS-guided individualized treatment recommendation would likely not be done when implemented in routine care	4
Flexibility in adherence	How different is the flexibility in how participants must adhere to the intervention and the flexibility likely in usual care?	Similar adherence to treatment and RR-TB management as in usual care	5
Follow up	How different is the intensity of measurement and follow-up of participants in the trial and the likely follow-up in usual care?	In addition to routine monthly sputum samples, sputum samples are collected at weeks 2, 3, 5 and 6 for *Mtb* culture to assess the primary outcome. Data is also collected on quality of life and costing for a health economics assessment from the patient perspective	4
Primary outcome	To what extent is the trial's primary outcome directly relevant to participants?	Time to culture conversion is a proxy for the patient-relevant outcomes of survival, cure and absence of relapse	3
Primary analysis	To what extent are all data included in the analysis of the primary outcome?	The analysis will be performed with all available data using an intention-to-treat approach	5

^a^Scored from 1 to 5 using a 5-point scale: 1 = very explanatory, 3 = equally pragmatic and explanatory, 5 = very pragmatic

## Methods: participants, interventions and outcomes

### Study setting {9}

The trial is embedded in the MDR-TB care infrastructure of the Lejweleputswa, Thabo Mofutsanyane, Mangaung Metro, and Fezile Dabi districts of the Free State Province of South Africa. Initial RR-TB treatment initiation, patient management, treatment monitoring and adherence monitoring are performed under routine conditions by the district MDR-TB teams that consist of an MDR-TB coordinator, NIMDR (Nurse-Initiate and Manage Drug-resistant TB) trained nurses and/or MDR-TB clinician(s) according to Department of Health (DoH) guidelines [40]. Only those drugs approved by South African DOH for TB treatment at standard dosing are used for trial participants. All decisions regarding clinical management are the authority of the DoH health care providers, regardless of participant allocation to the WGS or SOC arm.

### Eligibility criteria {10}

All adults (≥18 years) diagnosed with RR-TB not yet on RR-TB treatment are eligible unless they have complicated RR-TB disease (TB meningitis or TB of the bone), extrapulmonary TB without pulmonary involvement, refuse any RR-TB treatment, are unable to give informed consent, or reside far removed (>2-h drive) from the location of study field staff.

### Who will take informed consent? {26a}

Written informed consent from potential trial participants will be obtained in person by trained research staff.

### Additional consent provisions for collection and use of participant data and biological specimens {26b}

Additional consent is requested for storage for a minimum of 10 years of the *Mycobacterium tuberculosis* cultures and sputum samples.

## Interventions

### Explanation for the choice of comparators {6b}

The comparator is the standard of care RR-TB management as recommended by the South African national DoH guidelines [40]. All participants randomized to the control arm should initiate a short or long injection-free RR-TB treatment regimen upon RR-TB diagnosis. The care provider can individualize the treatment based on first- and second-line LPA and/or phenotypic DST results. Specifically, according to the guidelines, the dose of INH should be lowered to 300 mg daily in patients with INH-susceptible TB. When mutations in both *katG* and *inhA* are present or when FQ resistance is detected, patients should be switched to a long regimen or start the BDQ + LZD + DLM regimen. Patients with resistance to LZD, CFZ and/or BDQ should start a rescue regimen that is recommended by the Provincial Clinical Advisory Committee.

### Intervention description {11a}

**Fig. 1:**
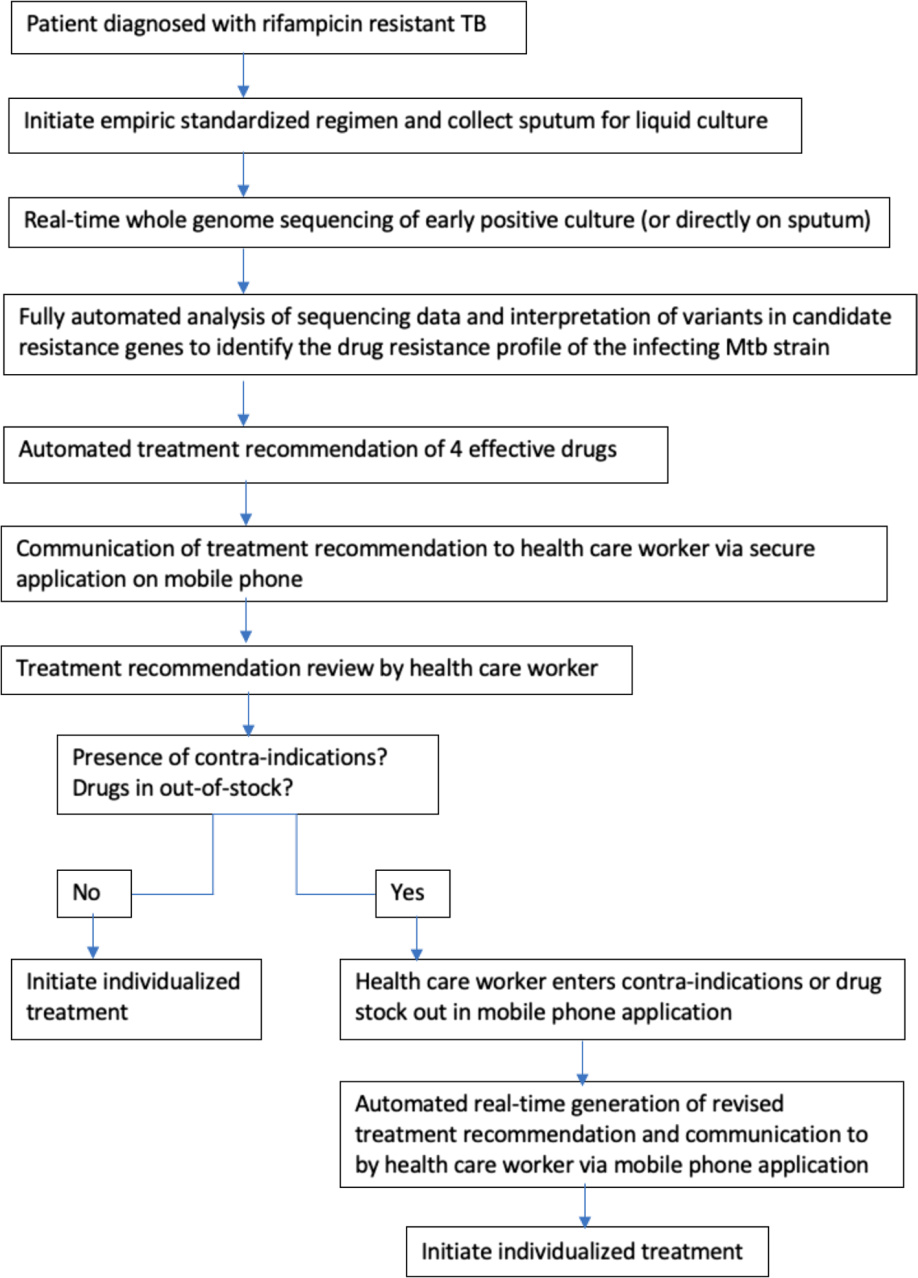
Strategy for whole genome sequencing-guided drug resistance profile determination and automated individualized treatment recommendation

The intervention consists of the use of *Mtb* WGS for determining the drug-resistance profile and automated individualized treatment recommendation of the optimal 4-drug regimen [34], with communication of the recommended regimen to the health care worker via a mobile phone app (Fig. 1).

All participants randomized to the intervention arm start a short or long injection-free RR-TB treatment regimen upon RR-TB diagnosis [40]. WGS is performed in real-time on DNA extracted from early positive Mycobacterium Growth Indicator Tube (MGIT) culture isolates of sputum specimens collected before the start of RR-TB treatment. In a sub-group of patients, culture-free WGS directly on DNA extracted from sputum specimens will be performed to evaluate the effect of a shorter turn-around time. An Illumina MiniSeq instrument is used for sequencing and WGS data are analysed using the compleX Bacterial Samples (XBS) bioinformatics pipeline [41]. Based on the WGS-derived drug resistance profile, the treatment recommender ranks all possible drug regimens for an individual patient. Regimens are scored based on the quantitative drug and regimen features including bactericidal and sterilizing activity, propensity to acquire resistance, toxicity, mechanism of action, route of administration, antagonistic and synergistic interactions, and cost. After taking into account clinical data (presence of contraindications, drug toxicity) and health systems data (drug stock out), the highest ranked 4-drug individualized treatment regimen is recommended for 6 months [34]. When healthcare workers question the WGS-guided treatment recommendation, a central clinical committee (CCC) of international experts in RR-TB treatment and clinical microbiology provides evidence-based guidance to the care provider.

To ensure the safety of participants randomized to the intervention, standard-of-care diagnostics are also performed and can be used to guide treatment when WGS results are not available due to poor quality sequencing reads or in case of negative cultures. In addition, phenotypic DST is performed for the drugs included in the WGS-guided individualized treatment regimen to identify possible errors in WGS-based resistance calling.

### Criteria for discontinuing or modifying allocated interventions {11b}

Criteria for discontinuing or modifying the allocated intervention for a given trial participant include the presence of a discordant phenotypic-genotypic drug resistance profile that suggests the administration of an ineffective treatment regimen or lack of culture conversion 4 months after initiation of the RR-TB treatment regimen.

### Strategies to improve adherence to interventions {11c}

In line with the design of a pragmatic trial, no strategies are implemented to improve adherence beyond those of routine care to improve adherence to RR-TB treatment. Participants in both trial arms will be phoned to remind them of the date of collection of four study sputum samples.

### Relevant concomitant care permitted or prohibited during the trial {11d}

Trial participation will not require alteration to usual RR-TB care pathways except for the recommendation of the individualized RR-TB treatment regimen in the intervention arm. Any concomitant care interventions are allowed during the trial.

### Provisions for post-trial care {30}

There is no anticipated need for ancillary and post-trial care. The trial is insured for compensation to those who may suffer harm from trial participation.

### Outcomes {12}

The primary effectiveness outcome is the bacteriological response to treatment measured as serial time to liquid culture positivity of sputum samples collected at baseline, weeks 2, 3, 4, 5, 6 and 8 and monthly thereafter. Secondary effectiveness outcomes include cure rate, treatment success rate, relapse rate (recurrence of RR-TB disease) and TB free survival rate in the first 12 months following RR-TB treatment completion. The outcome measure for the feasibility assessment is the number of patients in whom WGS is not successful (due to no *Mtb* growth, culture contamination or failed WGS) and the proportion of participants in whom the treatment recommendation is not followed. For the health economics assessment outcome, unit diagnostic and treatment costs for both arms will be estimated to calculate the total health system cost. The safety outcome is the proportion of participants who experience a serious adverse event.

### Participant timeline {13}

**Fig. 2:**
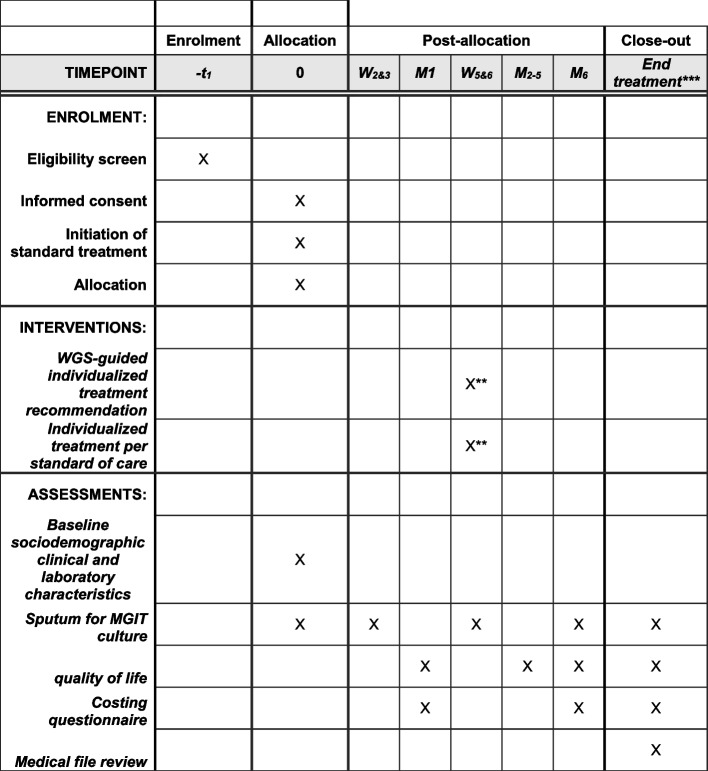
Schedule of screening, enrolment, interventions, and assessments. *Assessments listed are limited to the study-specific assessments. In addition to these, several assessments are performed on all study participants as per standard of care procedures. W, week; M, month; MGIT, Mycobacteria Growth Incubator Tube. ** Individualization of treatment occurs when drug susceptibility test results become available (whole genome sequencing for the experimental arm; line probe assays and phenotypic drug susceptibility test for standard of care arm). This is expected to occur a median of 5 to 6 weeks after the start of treatment. ***Closeout is performed at month 6 for patients who receive a 6-month treatment regimen

An overview of the screening, enrolment, participant timeline and schedule of assessments is provided in Fig. 2.

### Sample size {14}

The sample size was simulated for the primary objective using a non-linear mixed effect model. We re-calibrated the model described by Svensson and Karlsson [42] using RR-TB patient data from the Free State province to account for differences in study populations. We hypothesize that the intervention will reduce the mean half-life of mycobacterial load (βexp parameter in the model) by 28%. This would correspond to a difference in median time to culture conversion of 4 weeks (12 vs. 16 weeks). Given the equal balance across arms, and a significance level of 0.05, a total sample size of 173 individuals is needed to achieve 90% power to detect the hypothesized effect size. To account for negative or contaminated cultures at baseline (expected 16%) and death or loss to follow-up prior to achieving culture conversion (expected 21%), the total sample size was set at 248 participants.

### Recruitment {15}

All newly diagnosed RR-TB patients are notified to the study team either by central data warehouse notification of RIF-resistant cases or through communication by the district MDR-TB team. A district MDR-TB team member briefly informs the patient about the trial. If interested, the patient is referred to a study staff member for informed consent.

## Assignment of interventions: allocation

### Sequence generation {16a}

Randomization 1:1 to the WGS or control arm is automated and performed by Randomizer, a secure dedicated internet site [43].

### Concealment mechanism {16b}

Randomization is web-based and performed in real-time which guarantees unbiased allocation and eliminates the need for a concealment mechanism.

### Implementation {16c}

Randomization is web-based [43]. Minimization is implemented to obtain balanced groups by strata of factors associated with time to culture conversion (baseline mycobacterial load as determined by Xpert Ct value or smear microscopy grading), risk of mortality (age, diabetes, HIV status and if HIV positive whether the viral load is suppressed or not) and history of prior treatment with second-line drugs.

## Assignment of interventions: blinding

### Who will be blinded {17a}?

Participants are blinded to allocation to avoid any influence on adherence or reporting of adverse events by trial arm. Health care providers are not blinded to allocation but are blinded to the routine DST results for patients randomized to the WGS arm (unless required for patient safety) and to WGS results for patients randomized to the control arm.

### Procedure for unblinding if needed {17b}

Because the trial is single-blinded, where only the patient is blinded, we do not anticipate any circumstances under which unblinding is needed during the trial.

## Data collection and management

### Plans for assessment and collection of outcomes {18a}

The date of liquid sputum culture positivity is extracted from the MGIT instrument. Clinical and laboratory patient data is captured from the patient’s medical file, the South African DoH DR-TB treatment paper file and/or the Electronic Drug-Resistant Tuberculosis Register [44] (EDRWeb), and the NHLS laboratory Track Care system. Data extracted include TB treatment history, co-morbidities, HIV-related information, chest X-ray, sputum results (Xpert, LPA, smear microscopy), relevant laboratory test results, audiological screening, electrocardiogram, initial RR-TB regimen and drug doses, regimen adjustments, adherence, concomitant medications, toxicity, and treatment outcomes. The phenotypic drug resistance profile of the participant’s *Mtb* isolate is obtained by standard phenotypic drug resistance tests. The genotypic drug resistance profile is obtained through standard DNA extraction and sequencing procedures. Raw sequencing data will be retrieved from the Illumina Minseq instrument and analysed using the XBS bioinformatics pipeline [41]. Any failures encountered during the laboratory or bioinformatic analytic steps of the determination of the drug resistance profile will be entered in the electronic study database. Information on the prescription of WGS-guided individualized RR-TB treatment regimens will be captured through the secure treatment recommender web app. For the health economics assessment outcome, data on patient socioeconomic status, household assets, illness, and treatment-related costs, is collected by administering a questionnaire (adapted from a validated tool [45]) in the first month, after 6 months, and at the end of treatment for patients receiving more than 6 months of treatment. Data on quality of life is collected monthly using the EQ-5D-5L questionnaire [46]. Serious adverse events (SAEs) data will be captured from the SA DoH Pharmacovigilance form [17] and extracted from EDRWeb [44] and graded by an independent physician.

### Plans to promote participant retention and complete follow-up {18b}

Given the pragmatic design, there are no plans to promote participant retention beyond those implemented by the routine RR-TB care program.

### Data management {19}

Data will directly be entered in the electronic Research Electronic Data Capture (REDCap) database [47], which is developed to ensure data security and promote data quality through real-time data entry validation for data types and range checks. Source data collected as printouts such as pictures of the line probe assays will be captured in pdf format and stored in the REDCap database. Similarly, a picture of the signed informed consent form is uploaded in the electronic database. The REDCap database system employs various methods to protect and secure the data stored in the database.

### Confidentiality {27}

We use REDCap [47] to protect the confidentiality of personal data before, during, and after the trial. REDCap is a secure, web application designed to support data capture for research studies. It includes a full audit trail, user-based privileges, and de-identified data export mechanisms to statistical packages. Access to specific study data in REDCap will be restricted to the relevant members of the study team with authentication through password credentials.

### Plans for collection, laboratory evaluation and storage of biological specimens for genetic or molecular analysis in this trial/future use {33}

Sputum samples will be collected for laboratory evaluation and storage for genetic analyses of *Mycobacterium tuberculosis* in the current trial and for future use in ancillary studies.

## Statistical methods

### Statistical methods for primary and secondary outcomes {20a}

The primary analysis will be an intent-to-treat analysis. A non-linear mixed-effects model that includes a longitudinal representation of mycobacterial load, probability of bacterial presence in a sputum sample, and a time-to-event model to describe the changes in time to culture positivity in liquid media will be used to compare the bacteriological response between the two trial arms [42]. The feasibility of the WGS strategy will be determined based on the proportion of participants in whom WGS is not successful and the proportion of participants in whom the recommended treatment regimen is not prescribed. For the health economic evaluation, the mean incremental costs incurred by patients and the health care system for the WGS versus the control arm will be estimated. For evaluation of safety, we will compare the SAE rate during treatment between the two arms.

### Interim analyses {21b}

An interim analysis estimating the SAE rate will be performed when 50% of participants (n=124) are enrolled in the study and reviewed by the data safety monitoring board. The trial will be stopped if the rate of SAEs is significantly greater in the WGS arm as compared to the SOC arm.

### Methods for additional analyses (e.g., subgroup analyses) {20b}

Adjusted analyses will be performed to correct for possible differences in patient characteristics between groups. Subgroup analyses may be performed by drug resistance profile categories.

### Methods in analysis to handle protocol non-adherence and any statistical methods to handle missing data {20c}

The extent of missing data and patterns of missingness will be assessed once all study participants have completed the study. If needed, multiple imputation methods will be used and sensitivity analyses, including complete case analysis, will be conducted to evaluate the impact of missing data on study findings.

### Plans to give access to the full protocol, participant-level data and statistical code {31c}

We will abide by the requirements regarding making the underlying data and statistical code available set by the journal where we publish the results of the main trial outcome results. If not required by the journal, the de-identified dataset generated and analysed as well as the associated statistical codes will be made available after the publication of the main trial outcome results upon request from the corresponding author. Raw sequencing data will be submitted to a public sequence repository (European Nucleotide Archive) as part of the publication process.

## Oversight and monitoring

### Composition of the coordinating centre and trial steering committee {5d}

Several committees monitor the trial. The trial coordinator and field workers of the Clinical Research Organization (Aurum Institute) hold team meetings twice a week to provide day-to-day support for the trial. The central steering committee consists of 5 senior investigators. The central steering committee holds weekly meetings (also attended by the trial coordinator, four laboratory staff and three field workers) to solve any problems that arise during the implementation of the trial. The central clinical committee meets upon request to discuss potential patient safety issues that occur, such as phenotypic-genotypic discordance or failure to achieve culture conversion by week 16 of RR-TB treatment. The central microbiological committee meets upon request to discuss the interpretation of variants identified by WGS in drug resistance candidate genes that are not classified by the WHO catalogue of mutations in *Mycobacterium tuberculosis* [20]. When WGS data indicate possible BDQ resistance, the Free State Provincial Clinical Advisory Committee will be consulted to select the optimal individualized treatment.

### Composition of the data monitoring committee, its role and reporting structure {21a}

The data safety monitoring board (DSMB) consists of three experts in clinical trials and clinical management of drug-resistant TB. The members are independent of the sponsor and the clinical trial team and have no competing interests. The primary role of the committee is to safeguard the interests of the study participants by evaluating the difference in SAE rate between the two arms of the trial. The DSMB will meet when 50% of all participants have been enrolled and whenever required. The DSMB may also be asked to review factors external to the study such as scientific or therapeutic developments that may impact participant safety. Details about the committee are stated in the DSMB charter.

### Adverse event reporting and harms {22}

All SAEs reported to the study staff are reported to Free State University ethics committee and the South African Health Products Regulatory Authority. Because this is a pragmatic trial, all adverse events are managed by the physician in charge of the patient’s care.

### Frequency and plans for auditing trial conduct {23}

All data entered in the electronic database is audited weekly by the team of the principal investigator, independent of the sponsor. The trial conduct is not audited by a team that is independent of the investigators.

### Plans for communicating important protocol amendments to relevant parties (e.g., trial participants, ethical committees) {25}

Important protocol modifications will be communicated to the relevant parties, which may include the central clinical committee, data safety monitoring board, relevant ethics committees, South African Health Products Regulatory Authority, trial registries, and the sponsor.

### Dissemination plans {31a}

The investigators will communicate trial results to the provincial (Free State Province) and national (SA) DoH and the scientific community via presentations at national and international conferences. Results will be published in peer-reviewed scientific journals. De-identified data will be shared at the time of publication as per journal requirements or will be made available upon request.

## Discussion

WGS holds the promise to be a transformative technology in clinical microbiology, especially for slow-growing bacteria such as *Mtb*. While several low TB burden countries have successfully implemented WGS for routine DST, the integration of WGS into routine laboratories in high TB burden countries should be carefully evaluated before the capital investment, operational costs and human resource development required for such endeavour can be justified. Public health policymakers increasingly demand context-specific real-world evidence before implementing a novel intervention. This real-world evidence is most often obtained through observational studies, which are prone to biases. An explanatory RCT could provide an unbiased answer to the question of whether WGS-guided individualized treatment improves time to culture conversion under ideal conditions, not under real-life conditions. We designed a pragmatic RCT to answer the question whether of WGS-guided automated individualized treatment recommendation for RR-TB is effective under routine conditions in high-burden, low-resource settings. The pragmatic trial is designed to provide unbiased real-world evidence by maximizing generalizability while maintaining internal validity.

Several limitations to the trial should be noted. First, while the most important clinical endpoint is 2-year relapse free cure, the lengthy study duration required for this endpoint brings obvious challenges such as high cost and risk of high loss to follow-up rates. We therefore opted to employ a surrogate endpoint and replaced the clinical endpoint with a biomarker. We acknowledge that the surrogate endpoint may not be an accurate indicator for how effective the intervention is. Second, the WHO catalogue of mutations [20] is not exhaustive and knowledge on resistance-causing mutations is incomplete, especially for the new and repurposed drugs. This may reduce the effectiveness of WGS for guiding RR-TB treatments when newer regimens rely mainly on these drugs. Third, because culture-free WGS is still in its infancy, WGS will continue to rely on early positive liquid cultures in the near future. Finally, other molecular technologies are being developed and new treatment regimens are being evaluated. In 2021, the WHO has endorsed the Xpert MTB/XDR, which allows rapid identification of resistance to FQ but does not assess the resistance profile to other drugs included in most of the current and novel RR-TB treatment regimens [19]. The targeted deep-sequencing assay Deeplex Myc-TB can be used directly on sputum specimens with relatively high mycobacterial load for the prediction of resistance to 11 anti-tuberculous drugs/drug classes when investigating 18 of the 50 candidate resistance genes. Using phenotypic DST as the reference, high specificity (≥91%) was achieved for all drugs; sensitivity ranged from 54% for STM to 98% for RIF but could not be estimated for LZD and BDQ [48]. New standardized treatment regimens such as BDQ, Pa, LZD and moxifloxacin (BPaLM) mostly rely on the use of new drugs, making the regimen less prone to amplification of resistance [49]. The role of WGS-directed individualized treatment should thus be evaluated relative to these new developments.

In conclusion, as Cox et al. wrote, “seeking the highest quality of care in all settings should be an ethical imperative for treatment of drug-resistant tuberculosis” [50]. Without individualized treatment guided by drug resistance profiles, routine use of any novel empiric regimen in high RR-TB burden settings will over time result in the emergence of resistance. WGS to determine the complete *Mtb* drug resistance profile has already replaced phenotypic DST in several high-income countries, where precision medicine based on the individual patient and pathogen characteristics is already established. In low- and middle-income countries, WGS is not yet used for clinical care in part due to technical challenges and difficulties in analyzing WGS data and translating WGS information into individualized RR-TB treatment regimens. The use of an individualized treatment recommendation system could overcome this hurdle by automating the individualization of treatment regimen selection and guiding clinicians independent of their level of expertise. Hopefully, quantifying the effectiveness of WGS-guided individualized treatment recommendation for RR-TB treatment, evaluating the feasibility thereof, and assessing health economics from both the health system and patient perspective will play a critical role in answering the question of whether WGS should be recommended for the management of RR-TB in high TB-burden, low-resource settings.

### Trial status

The trial with protocol version 6.0 (June 9, 2021) was registered on the clinical trials.gov register with the number NCT05017324 on August 23, 2021. The first patient was recruited on September 23, 2021, and is expected to be completed by the end of 2022.

## Data Availability

The final de-identified trial dataset will be made available at the time of publication of the main study findings if required by the journal publication policy or via request to the corresponding author if not required by the journal’s publication policy. The investigators are not bound to any contractual agreements that limit such access.

## Abbreviations

AMK: Amikacin
BDQ: Bedaquiline
CCC: Central clinical committee
CFZ: Clofazimine
Ct: Cycle threshold
DLM: Delamanid
DNA: Desoxyribonucleic acid
DoH: Department of Health
DR-TB: Drug-resistant tuberculosis
DSMB: Data safety monitoring board
DST: Drug susceptibility test
EDRWeb: Electronic Drug-Resistant Tuberculosis Register
EMB: Ethambutol
ETO: Ethionamide
FQ: Fluoroquinolone
HIV: Human immunodeficiency virus
INH: Isoniazid
LFX: Levofloxacin
LPA: Line probe assay
LZD: Linezolid
MDR-TB: Multidrug-resistant tuberculosis
MFX: Moxifloxacin
MGIT: Mycobacterium growth indicator tube
Mtb: Mycobacterium tuberculosis
NIMDR: Nurse-Initiate and Manage Drug-resistant TB
NGS: Next-generation sequencing
Pa: Pretonamid
PCR: Polymerase chain reaction
PZA: Pyrazinamide
RCT: Randomized clinical trial
RCT: Randomized controlled trial
REDCap: Research Electronic Data Capture
RIF: Rifampicin
RR-TB: Rifampicin-resistant tuberculosis
SAE: Serious adverse event
STM: Streptomycin
TB: Tuberculosis
TZD: Terizidone
WGS: Whole-genome sequencing
WHO: World Health Organization
XDR-TB: Extensive drug-resistant.
